# Appetite- and Weight-Regulating Neuroendocrine Circuitry in Hypothalamic Obesity

**DOI:** 10.1210/endrev/bnad033

**Published:** 2023-11-29

**Authors:** Hoong-Wei Gan, Manuela Cerbone, Mehul Tulsidas Dattani

**Affiliations:** Department of Endocrinology, Great Ormond Street Hospital for Children NHS Foundation Trust, Great Ormond Street, London WC1N 3JH, UK; Genetics & Genomic Medicine Research & Teaching Department, University College London Great Ormond Street Institute for Child Health, 30 Guilford Street, London WC1N 1EH, UK; Department of Endocrinology, Great Ormond Street Hospital for Children NHS Foundation Trust, Great Ormond Street, London WC1N 3JH, UK; Genetics & Genomic Medicine Research & Teaching Department, University College London Great Ormond Street Institute for Child Health, 30 Guilford Street, London WC1N 1EH, UK; Department of Endocrinology, Great Ormond Street Hospital for Children NHS Foundation Trust, Great Ormond Street, London WC1N 3JH, UK; Genetics & Genomic Medicine Research & Teaching Department, University College London Great Ormond Street Institute for Child Health, 30 Guilford Street, London WC1N 1EH, UK

**Keywords:** obesity, appetite, hypothalamus, anorexigen, orexigen

## Abstract

Since hypothalamic obesity (HyOb) was first described over 120 years ago by Joseph Babinski and Alfred Fröhlich, advances in molecular genetic laboratory techniques have allowed us to elucidate various components of the intricate neurocircuitry governing appetite and weight regulation connecting the hypothalamus, pituitary gland, brainstem, adipose tissue, pancreas, and gastrointestinal tract. On a background of an increasing prevalence of population-level common obesity, the number of survivors of congenital (eg, septo-optic dysplasia, Prader–Willi syndrome) and acquired (eg, central nervous system tumors) hypothalamic disorders is increasing, thanks to earlier diagnosis and management as well as better oncological therapies. Although to date the discovery of several appetite-regulating peptides has led to the development of a range of targeted molecular therapies for monogenic obesity syndromes, outside of these disorders these discoveries have not translated into the development of efficacious treatments for other forms of HyOb. This review aims to summarize our current understanding of the neuroendocrine physiology of appetite and weight regulation, and explore our current understanding of the pathophysiology of HyOb.

## Graphical Abstract

**Graphical abstract:**
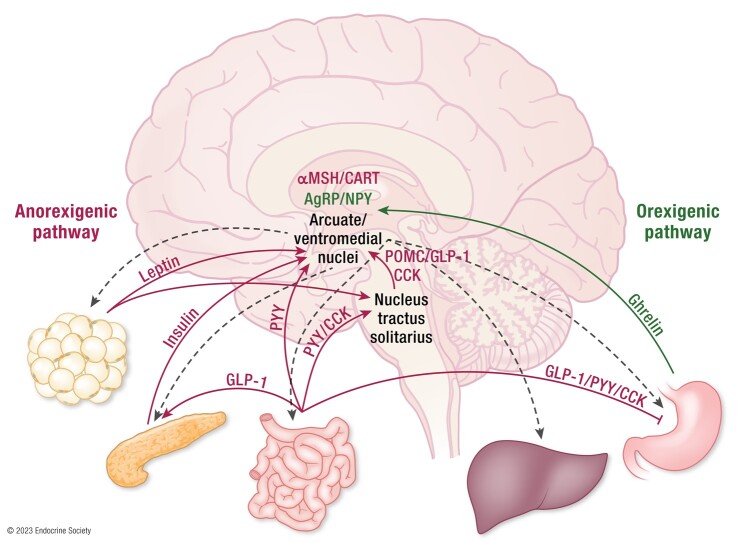

**Essential Points**

- Hypothalamic obesity is characterized by intractable weight gain in the presence of congenital hypothalamic dysfunction or acquired hypothalamic damage
- Our current understanding of the complex neuroendocrine circuitry regulating our appetite and weight has advanced significantly over the last 3 decades, but remains incomplete
- Appetite and weight are maintained through the actions of orexigenic (eg, ghrelin, neuropeptide Y) and anorexigenic (eg, leptin, insulin) hormones, which interact through multiple neuroendocrine networks
- The development of targeted molecular treatments for monogenic hypothalamic obesity syndromes has revolutionized the landscape for these rare conditions
- However, treatment of congenital or acquired syndromic hypothalamic obesity is still lacking, due to our incomplete understanding of these circuits which are often redundant in nature

## Hypothalamic Obesity: A Historical Perspective

Hypothalamic obesity (HyOb) has been defined loosely as a “syndrome of intractable weight gain after any hypothalamic damage” (1). Despite only measuring about 0.7 cm^3^ on either side of the third ventricle (2), the hypothalamus is a critical region of the brain responsible for regulating 7 endocrine axes via its intimate connections to the pituitary gland, as well as appetite, metabolism, thirst, circadian rhythms, arousal, temperature, memory, and behavior. Therefore, damage or maldevelopment of this area can have widespread consequences on a variety of homeostatic mechanisms and unsurprisingly cause, in addition to obesity and appetite dysregulation (classically hyperphagia), panhypopituitarism, sleep disturbances, temperature dysregulation, and behavioral disorders (often termed as the “hypothalamic syndrome” when present in combination). The dense congregation of neurons in this area, the poor definition of the various hypothalamic nuclei, and the limited resolution of current neuroradiological techniques, however, has meant that our current understanding of the circuitry underlying these clinical manifestations remains incompletely understood. Experiments manipulating the various hypothalamic genes involved also often result in effects on multiple neuronal subtypes or even lethality, further complicating the task of deciphering their role in the development of this region (3, 4).

HyOb was first described independently in the early 1900s by the neurologists Joseph Babinski (5) and Alfred Fröhlich (6), who respectively described virtually identical cases of an adolescent girl and boy presenting with headaches, visual impairment, short stature, an increased body mass index (BMI) with centripetal fat distribution and pubertal delay, both of whom were found to have a hypothalamo-pituitary mass at postmortem. The association between the abnormal fat distribution and genital underdevelopment seen in these cases led to the Babinski–Fröhlich syndrome initially being described as “adiposogenital dystrophy” (7).

Over 100 years since its original description, our understanding of the pathophysiology of HyOb has advanced significantly, but remains incomplete. Early experiments in rodents demonstrated firstly that damage to the hypothalamus, but not the pituitary, was necessary for its development (8, 9). Subsequent more detailed work demonstrated that the ventromedial nucleus (VMN) within the hypothalamus was crucial for the regulation of weight and appetite, while the arcuate nucleus (ARC) and lateral hypothalamic area (LHA) were involved to a lesser degree (8, 10). Subsequent experiments also indicated that hyperphagia was not necessary for the development of obesity, as rats with bilateral VMN electrolytic lesions still became obese and hyperinsulinemic when entirely tube-fed, whereas their pair-fed counterparts did not (11). The first hint of the underlying molecular mechanism for HyOb appeared with the description of the spontaneous breeding of the *ob/ob* (obese) mouse at a ratio of 1:3 to the rest of the litter, suggesting an autosomal recessive inheritance, but achieving a weight 3 times that of its littermates by ten months of age (12).

However, it took over 40 years for the advent of molecular techniques to clear the path for the cloning of the *Ob* gene product, leptin—the first hormone linking the hypothalamus with the regulation of appetite and metabolism in humans (13). This was followed shortly after by the identification of the first monogenic cause of human obesity, congenital leptin deficiency (14), and its successful treatment with recombinant human leptin (15). Concurrently, a rapid cascade of discovery of other hormones and neuropeptides involved in the neuroendocrine regulation of appetite and weight was occurring, including brain-derived neurotrophic factor (BDNF) (16), neuropeptide Y (NPY) (17), polypeptide YY (18), glucagon-like peptide-1 (GLP-1) (19), cocaine- and amphetamine-regulated transcript (CART) (20), agouti-related peptide (AgRP) (21), and ghrelin (22). Insulin (23) and α-melanocyte stimulating hormone (αMSH) (24) had already been discovered decades earlier, but their role in appetite regulation in the hypothalamus was not appreciated until several years later (25, 26). Over the last 2 decades, the corresponding human obesity phenotypes for mutations in the genes encoding the above peptides have gradually been elucidated, further corroborating their role in appetite and weight homeostasis.

## Etiology of Hypothalamic Obesity

While HyOb is a relatively rare form of obesity, it is frequently seen in patients with both congenital and acquired disorders involving maldevelopment, dysfunction or damage to the hypothalamo-pituitary unit. Apart from the various “nonsyndromic” and syndromic genetic obesity syndromes described in further detail in “Monogenic ‘nonsyndromic’ hypothalamic obesity syndromes” and “Syndromic forms of obesity without hypothalamic structural defects,” HyOb is also a feature of various diseases affecting the structure of the hypothalamus. The archetypal congenital midline neurodevelopmental structural disorder of this region, septo-optic dysplasia (SOD) is a syndrome of maldevelopment of the optic pathways in combination with other midline forebrain defects and/or hypopituitarism (27), where up to 31% of patients develop obesity (28). Similarly, inexorable, extreme obesity is seen commonly in survivors of a wide variety of suprasellar midline brain tumors such as craniopharyngiomas (up to 77% (29)), low-grade optic pathway gliomas (50-75% (30, 31)), hypothalamic hamartomas (28-59% (32, 33)), pituitary adenomas (39% (34)), and intracranial germinomas (14% (35)). Other acquired causes of HyOb include traumatic brain injury (36), infiltrative causes such as Langerhans cell histiocytosis (37), and inflammatory causes such as hypophysitis (38).

In this latter group of patients, HyOb can be a consequence of both tumor and/or targeted treatments to the area, such as surgery or radiotherapy. An analysis by Lustig et al (2003) (39) demonstrated that in a cohort of pediatric brain tumor survivors, HyOb was associated with hypothalamic tumor involvement, tumor histologies frequently presenting in the suprasellar midline (such as craniopharyngiomas), radiotherapy to the hypothalamus exceeding 51 Gy, extensive hypothalamic surgery, and/or the presence of any other hypothalamo-pituitary endocrinopathy, as well as an age of <6 years at diagnosis. Therefore, despite the rarity of HyOb compared with obesity in the general population (henceforth termed “common obesity”), a cohort of patients with both congenital and acquired forms of the disorder is rapidly accruing. The severity of obesity and its attendant sequelae, as well as a current lack of effective treatment options means that the financial burden on families and clinical services is significant, and an understanding of its underlying pathophysiology is becoming ever more crucial.

## Physiology of Normal Appetite and Weight Regulation

The discovery of the monogenic “nonsyndromic” obesity disorders (see “Monogenic ‘nonsyndromic’ hypothalamic obesity syndromes”) in both animals and humans has helped elucidate the complex interplay between appetite-suppressing (anorexigenic) and appetite-stimulating (orexigenic) hormones circulating between the hypothalamus and peripheral tissues. The hypothalamic–gut–adipose tissue circuit can be thought of as consisting of an “afferent” arm where peripheral tissues signal to the hypothalamus about the body's metabolic status, and an “efferent” arm where the hypothalamus signals back to the peripheral tissues via the autonomic nervous system to maintain homeostasis of the human body's energy balance (1, 40-42).

### Anorexigens

#### Leptin

Leptin is a 167 amino acid protein encoded by the *LEP* gene on chromosome 7q32.1. As previously discussed, the first clue alluding to the existence of leptin arose from the spontaneous breeding of the *Ob/Ob* mouse (12), which subsequently led to the cloning of the gene and the identification of its protein in mice and humans (13). Shortly after its discovery, the *Db/Db* mouse was also described (43), and cloning of the corresponding gene causing this phenotype, the leptin receptor (*LEPR*), followed (44). The human phenotypes of congenital leptin deficiency and congenital leptin receptor deficiency further cemented the role of leptin in appetite and weight homeostasis (14, 45).

Leptin is synthesized predominantly in white adipose tissue (13), and plasma concentrations are unsurprisingly strongly positively correlated with BMI (46). Serum leptin concentrations are higher in women than in men, but are not related to resting energy expenditure, indicating that its main effect is on appetite over metabolism (47). Indeed, serum leptin has been shown to increase significantly in response to both acute and chronic overfeeding, demonstrating that leptin secretion is not purely a function of fat mass accumulation (48). Contrastingly, leptin concentrations fall with fasting (49). Leptin is also secreted by CD4+ T cells (50) and chondrocytes (51), where it acts as a cytokine. Contrastingly, *LEPR* is expressed on a wide variety of tissues apart from the hypothalamus and undergoes alternative splicing to generate 4 isoforms—HLR-5, HLR-15, HLR-67, and HLR-274 (52-54). The longest isoform, HLR-274, is responsible for the effects of leptin on energy homeostasis, with expression in the ARC, VMN, LHA, dorsomedial nucleus (DMN), and paraventricular nucleus (PVN) of the hypothalamus (55, 56), but it is also the most rapidly downregulated and transported to lysosomes for degradation, thus causing the syndrome of functional leptin resistance seen in obesity (53). The ARC appears to be the predominant hypothalamic region responsible for leptin's anorexigenic effects, as rats with a nonfunctioning ARC do not exhibit a reduction in food intake and weight loss in response to centrally administered leptin (57, 58).

Binding of leptin to HLR-274 causes signal transduction via multiple secondary messengers, including the Janus kinase/signal transducer and activator of transcription (JAK/STAT) and the phosphoinositide 3-kinase/protein kinase B/Forkhead box protein 1 (PI3K/PKB (AKT)/FOXO1) pathways (59). Through the former, leptin increases the transcription of proopiomelanocortin (POMC), the precursor of the major anorexigen αMSH (60), while the latter pathway is common to both leptin and insulin signaling, with both hormones stimulating POMC neurons, and leptin additionally inhibiting AgRP neurons (61, 62) Furthermore, leptin suppresses the FOXO1-stimulated expression of NPY and AgRP and FOXO1-inhibited expression of POMC (63, 64). The role of leptin in regulating POMC vs AgRP/NPY secretion is further supported by the coexpression of leptin receptors on both of these neuronal subsets (65, 66).

#### Insulin

Insulin is a 51 amino acid protein encoded by the *INS* gene on chromosome 11p15.5 and consists of 2 polypeptide chains (A and B) linked by disulfide bonds. Both of these originate from the same *INS* gene product, proinsulin, which is cleaved by endopeptidases to form mature insulin in the β cells of the pancreas (67), which acts on the insulin receptor (INSR), present on a wide variety of tissues including the hypothalamus (68-70).

The discovery of insulin as a treatment for diabetes mellitus has been known for decades and is a longstanding historical landmark in the field of endocrinology (23), but its role in hypothalamic signaling and the regulation of appetite has only been recently deciphered (69-71). Intracerebroventricular and hypothalamic infusions of insulin have been shown to cause sustained reductions in food intake and body weight in baboons and rats respectively, with the effect being negated by the presence of anti-insulin antibodies in the VMN (72, 73). Reduction of *Insr* expression in the hypothalamus by antisense oligonucleotides in rats, and conditionally knocking out *INSR* in neuronal tissues in mice both result in increased appetite and weight gain (74, 75). Insulin has been shown to inhibit hypothalamic *Npy* and *Agrp* expression, and to increase *Pomc* neuronal activation in rodent models in concert with leptin through PI3K/PKB (AKT)/FOXO1 signaling (61, 63, 76-79).

In insulin-dependent diabetes mellitus (IDDM), an increased appetite occurs with both hyperglycemia and hypoglycemia. Rats and humans with IDDM have reduced adiposity and consequent leptin deficiency, which is reversible with insulin supplementation (80, 81). Similarly, adipose tissue-specific *Insr* conditional knockout mice display the opposite phenotype to hypothalamic conditional knockouts, with a low fat mass, protection against age-related and HyOb, and reduced glucose intolerance (82). This is because insulin is required for fat deposition in adipocytes. Hyperglycemic hyperphagia is therefore mediated by leptin deficiency, with leptin replacement in untreated rats with IDDM ameliorating the phenomenon of diabetic hyperphagia, despite the diabetes remaining untreated (83). Conversely, clinicians treating IDDM will also be aware that appetite increases with insulin replacement. The apparent dichotomy of these insulin effects may be explained by the effect of insulin on glucose production—insulin induces hypoglycemia which in turn increases appetite rather than via a direct effect of insulin in itself. This principle was demonstrated elegantly in experiments by Booth (1968) (84), where concurrent administration of intrahypothalamic glucose and subcutaneous insulin did not lead to an increased appetite in rats.

#### The POMC/αMSH/CART system

POMC is a 267 amino acid polypeptide encoded by the *POMC* gene on chromosome 2p23.3. This protein is a precursor of 5 separate peptide hormones—adrenocorticotropic hormone (ACTH), α-, β-, and γ-melanocyte stimulating hormones, and β-endorphin—which are synthesized by a series of post-translational proteolytic cleavage steps largely governed by the serine proteases prohormone convertases 1 and 2 (PC1 and PC2) (85-87). *POMC* is expressed in the hypothalamus, anterior and intermediate lobes of the pituitary gland, nucleus tractus solitarius (NTS), immune system, and skin, and the degree of proteolytic cleavage is site dependent (88). For instance, corticotrophs in the anterior pituitary only produce PC1 and are therefore only able to synthesize ACTH from POMC, whereas melanotrophs in the intermediate pituitary in rodents and skin cells in humans produce both PC1 and PC2 and are therefore able to synthesize αMSH, which stimulate melanogenesis (89, 90). In the central nervous system (CNS), *Pomc* expression is restricted to the ARC and NTS, both of which exhibit PC1 and PC2 activity, producing α-, β-, and γMSH in the former, and αMSH and β-endorphin in the latter (91-93).

In mice, acute activation of NTS POMC neurons causes a short-term reduction in food intake with no change in body weight, while chronic activation of ARC POMC neurons is required to lead to the same effect, but additionally results in a reduction in weight. Correspondingly, it is ablation of ARC POMC, not NTS POMC neurons, which is required for the development of obesity, increased fat mass and glucose intolerance (94), with the former neurons being stimulated by leptin (95), while the latter are stimulated by cholecystokinin (CCK) and inhibited by opioids (96). Overfed rats have a marked increase in ARC POMC expression which results in a negative feedback loop reducing appetite; the effects of which are reduced in the presence of a melanocortin 3/4 receptor (MC3R/MC4R) antagonist (97). In humans, homozygous *POMC* mutations cause clinical features involving deficiency of all its derivative peptides, including severe obesity, adrenal insufficiency, and red hair (98).

The diversity of peptides generated from POMC is mirrored by the diversity of their G-protein–coupled melanocortin receptors (MCRs). MC1R, MC2R, and MC5R are responsible for skin pigmentation (melanocytes), ACTH induction of steroidogenesis (adrenal glands) and temperature regulation (widespread exocrine tissues) respectively (90, 99, 100). MC3R and MC4R both regulate appetite and weight, with MC4R being the predominant receptor present in the PVN, DMN, and LHA, but also in the cortex, thalamus, brainstem and spinal cord (101), while MC3R is restricted to the ARC (102). MC4R receptors on POMC neurons additionally appear to be responsible for increasing energy expenditure without an increase in food intake (103), and there is a suggestion that MC4R signaling in the PVN is important for integrating circadian light cues with glucose metabolism (104). *Mc4r^−/−^* knockout mice develop severe hyperphagia and obesity (105), and dominant mutations in *MC4R* have been shown to be the most common cause of monogenic obesity in humans (106, 107). Interestingly, double knockout *Mc3r^−/−^*/*Mc4r^−/−^* rats demonstrate even more severe hyperlipidemia and hyperglycemia than *Mc4r^−/−^* knockout rats, despite the fact that knocking out the *Mc3r* gene alone causes hypophagia and fat mass accumulation without necessarily becoming overweight (108-110).

Concurrently, leptin stimulates ARC CART hypothalamic neurons, the large proportion of which coexpress POMC/αMSH (111-113). CART is secreted as a 116 amino acid prepropeptide coded for by the *CARTPT* gene on chromosome 5q13.2, with CART being present not just in the hypothalamus (where it has been found in the PVN, supraoptic nucleus [SON], ARC, zona incerta, and anterior PVN), but also in the pituitary, nucleus accumbens, frontal cortex, midbrain and adrenal medulla (20, 114, 115). Like POMC, it is post-translationally proteolytically cleaved into shorter peptides which are biologically active (116). To date, however, the equivalent receptor for CART has yet to be identified. However, intracerebroventricular administration of CART in rats has been shown to inhibit food intake, even cancelling out the orexigenic effect of NPY (112, 117). ARC POMC/CART neurons have additionally been shown to project to the PVN and regulate the release of thyrotrophin-releasing hormone (TRH), and is 1 postulated mechanism by which they regulate metabolic homeostasis (118, 119). Other projections of POMC/CART neurons include the medial preoptic nucleus, DMN, LHA, and the ARC itself, all of which are involved in appetite and weight regulation (120, 121). *Cart^−/−^* knockout mice exhibit an increase in food intake and weight gain, and impaired glucose tolerance and insulin secretion through its direct impact on pancreatic β-cell function (122, 123). Similarly, heterozygous *CARTPT* mutations have been described in a family with severe obesity and reduced metabolic rates (124).

#### Brain-derived neurotrophic factor

BDNF is a 119 amino acid protein encoded by the *BDNF* gene on chromosome 11p14.1 (125). As a member of the nerve growth factor (neurotrophin) family, it has a major role in neuronal survival and neuroprotection, and is therefore widely expressed (126-129). In the rodent hypothalamus, *Bdnf* is expressed in the VMN, PVN, LHA, and anterior hypothalamus (130, 131). However, the role of BDNF in regulating appetite and weight has proven difficult to study due to its crucial role in the nervous system, with *Bdnf^−/−^* mice exhibiting early postnatal lethality (132, 133).

The first clue to the role of BDNF in appetite–weight homeostasis appeared in studies of heterozygous *Bdnf^+/−^* mice, where food intake and weight (particularly fat mass) were increased over time, and serotonergic transmission was dysregulated (130, 134). Intracerebroventricular infusion of BDNF in these mice caused significant weight loss (130). The role of serotonin (5-HT) in BDNF signaling, was supported by the effects of fluoxetine, which partially reduced the amount of food ingested by *Bdnf^+/−^* mice, while the role of BDNF as a neurotrophin in maintenance of 5-HT neurons was demonstrated by the gradual reduction in their number over time, from baseline normal quantities (134). Interestingly, in experiments by Kernie et al (2000) (130), subgroup analysis in the proportion of *Bdnf^+/−^* mice who gained weight more slowly demonstrated that these mice also exhibited a marked increase in locomotor activity compared with the *Bdnf^+/−^* mice who became frankly obese. The baseline hypothalamic expression of *Npy*, *Agrp, Cart*, and *Lepr* was not altered in brain conditional knockout mice, but *Pomc* expression was increased, and the leptin and NPY responses to starvation were abnormal (135). BDNF has also been shown to have hypoglycemic and anti-diabetic effects by improving insulin secretion and sensitivity (136). To date, only 1 disease-causing *BDNF* point mutation has been identified in humans in association with hyperphagia, obesity, hyperactivity and impaired cognitive function (137). Additionally, the obesity observed in the 11p13-14 contiguous gene deletion WAGRO syndrome (Wilms tumor, aniridia, genitourinary abnormalities, mental retardation, obesity) has been ascribed to *BDNF* haploinsufficiency (138).

BDNF acts through tyrosine kinase receptor B (TRKB) which is encoded by the *NTRK2* gene on chromosome 9q21.33. *NTRK2* is widely expressed across the brain and other tissues (139). Similar to the phenotype of *Bdnf^+/−^* heterozygotes, *Ntrk2* hypomorphic mice also show hyperphagia, increased fat accumulation and obesity (131). Administration of an MC4R agonist in the fasted state significantly increases *Ntrk2* expression in the VMN, indicating that the BDNF/TRKB system lies downstream of the POMC/αMSH/MC4R system (131). *NTRK2* heterozygous mutations have been described in human syndromes of obesity and developmental delay (140, 141).

#### Glucagon-like peptide-1

GLP-1 is 1 of 2 incretins secreted by the gut to stimulate insulin secretion in response to food (142). It is encoded by the same gene encoding glucagon, *GCG*, on chromosome 2q24.2, with glucagon, GLP-1 and GLP-2 being coded in sequential order and the entire polypeptide being synthesized as proglucagon, which is post-translationally proteolytically cleaved by PC1 and PC3 into its constituent peptides (143, 144). GLP-1 is a potent stimulant for insulin secretion, and is predominantly released by enteroendocrine L cells in response to nutrient intake to act on its corresponding receptor, GLP-1R (145-147). Additionally, GLP-1 is also produced in the NTS, with projections to the hypothalamus (148-150). In the brain, *GLP1R* is expressed in the cerebral cortex, SON, PVN, VMN, ARC, DMN, hippocampus, thalamus, caudate nucleus, putamen, and globus pallidus (149-151).

A specific phenotype associated with deleterious variants in *GCG* and *GLP1R* has yet to be defined in humans, although *Glp1r^−/−^* knockout mice gain less weight when exposed to a high-fat diet, and seem to be protected from insulin insensitivity (152). Cell-specific *Glp1r* mouse knockdown experiments in PVN and POMC neurons, however, suggest that GLP-1 appears to work independently of these networks, as these mice did not exhibit changes in food intake or weight (although PVN *Glp1r* knockdown mice showed a reduction in energy expenditure), and GLP-1 agonist administration still led to anorexia and improved glucose tolerance (153). Its effects may instead be predominantly mediated by modulation of orexigenic pathways, as intracerebroventricular administration of GLP-1 in rats attenuated NPY-induced food intake (149), but results are inconsistent and the hypothalamic pathways through which it exerts its effect remain to be fully elucidated (154).

Overall, however, most studies show that intracerebroventricular infusion of GLP-1 causes a marked reduction in food intake in rats, and, in the longer term, weight loss, by attenuating the increased orexigenic (NPY/AgRP) and reduced anorexigenic (POMC/CART) responses to fasting, independent of its effects on gastric emptying (149, 155-158). Additionally, GLP-1 induces c-*fos* expression in corticotrophin-releasing hormone (CRH) and oxytocin (OXT) neurons of the PVN and SON, both of which have anorexigenic effects (150). The role of GLP-1 originating from the NTS must also not be underestimated, as NTS-specific knockdown of the *Gcg* gene in rats resulted in hyperphagia, high-fat diet–induced weight gain and fat accumulation, and glucose intolerance (159). The action of GLP-1 is terminated rapidly by the actions of dipeptidyl peptidase-4 (DPP4), which is present in the capillaries of the intestinal mucosa (160). Administration of exogenous GLP-1 in humans causes increased satiety, reduced food intake, delayed gastric emptying, and improved glucose tolerance (161-164), with a recent meta-analysis of 25 studies showing an overall effect of weight loss (165). Lastly, GLP-1 concentrations rise along with PYY after bariatric surgery, and this rise has been postulated to contribute to the sustained weight loss observed (166, 167).

#### Cholecystokinin

CCK is a predominantly gut peptide hormone expressed in I cells throughout the small intestinal mucosa (apart from the terminal ileum) and is synthesized as a 95 amino acid propeptide encoded by the *CCK* gene on chromosome 3p22.1 (168-170). Pro-CCK is then proteolytically cleaved into peptides of multiple lengths, with CCK-33 being the predominant form found in human plasma and small intestine (171). Additionally, CCK is also expressed widely in the CNS, with high concentrations in the corpus striatum, hippocampus, hypothalamus, thalamus, basal ganglia, mesencephalon, and brainstem (particularly the NTS); in these regions, CCK-8 is predominant (172-175). In the NTS, neurons with the HLR-274 leptin receptor have been found to also contain POMC, glucagon and CCK, providing a link between these various appetite-regulating networks in the brainstem (174). Like many other appetite-regulating hormones, the differential expression of the various molecular forms of CCK in different tissues is governed largely by the expression of different PC enzymes (176).

CCK acts through its 2 receptors, CCKAR and CCKBR, with the former thought to be responsible for its anorexigenic effects in the brain (175, 177), despite CCKBR being the predominant form found in the CNS (178, 179). CCKAR is largely found peripherally (in the pancreas and stomach where it delays gastric emptying), with brain expression being restricted to the area postrema and NTS and, unlike CCKBR, has a high degree of specificity for CCK-8 (180-182). These areas are well-known to be deficient in the blood–brain barrier, therefore supporting the postulation that these receptors are responsible for the detection of satiety signals from the periphery, particularly when peripherally administered CCK-8 has been shown not to generally enter the cerebrospinal fluid space (183) Peripheral administration of CCK-8 has been shown to inhibit food intake in both animal models and humans, supporting this theory (184-186). Targeted CCK-8 injections in rats have shown that this effect is specific for the anterior hypothalamus, DMN, LHA, PVN, SON, and VMN as well as the NTS (187). CCK NTS neurons project to the PVN, as well as the VMN, DMN, and ARC, where its effects on MC4R neurons are mediated by CCK-8 on CCKAR (175). Conversely, administration of a CCKAR antagonist has been shown to increase the subjective sensation of hunger in men (177). In a spontaneous rat model of obesity, the Otsuka Long Evans Tokushima Fatty (OLETF) rat, *Cckar* is deleted, resulting in hyperphagia, obesity and noninsulin-dependent diabetes mellitus (188, 189). *CCKAR* variants and mutations have been reported to be associated with obesity in humans (190, 191).

#### Peptide YY and pancreatic polypeptide Y

PYY and PPY (both 36 amino acids) are members of the NPY class of neuropeptides, all of which are involved in regulating appetite and share sequence homologies (192). Both *PYY* and *PPY* genes are located on chromosome 17q21.31, approximately 10 kb apart, suggesting that these genes arose from a duplication event (193).

PYY, like GLP-1, is expressed in enteroendocrine L cells of the lower gastrointestinal tract where it is released postprandially, as well as the endocrine pancreas and enteric neurons, with smaller amounts in the adrenal gland, respiratory tract, pituitary gland and hypothalamus (where it is found in the PVN, ARC, and SON (194-197). Cleavage of PYY_1-36_ by DPP4 generates PYY_3-36_, which is able to cross the blood–brain barrier (198-200). This cleavage step is necessary for the activation of PYY as an anorexigen, as PYY_1-36_ has no impact on food intake in rats deficient in DPP4 (201).

The anorexigenic effects of PYY_3-36_ are mediated via the Y2 receptor which is encoded by the *NPY2R* gene on 4q32.1 and expressed in myenteric neurons in the gastrointestinal tract, the hippocampus, ARC, medial preoptic nucleus, NTS, area postrema, and the piriform cortex (202-204). Peripheral administration of PYY_3-36_ in rodents and humans results in inhibition of food intake and weight gain, but this effect is not seen in *Y2r^−/−^* mice (205). Appetite suppression is mediated via the ARC by reducing hypothalamic *Npy* expression and activating POMC neurons. An intact POMC/MC4R system, however, is not requisite for this effect as PYY_3-36_ is also anorexigenic in *Pomc^−/−^* and *Mc4r^−/−^* mice (206, 207). Importantly, although human obesity is associated with PYY deficiency, PYY_3-36_ retains its anorexigenic effects in this group, without the development of resistance like that seen with leptin (208, 209). Additionally, PYY_3-36_ has also been shown to activate catecholaminergic neurons in the area postrema and NTS in rats (209). However, development of longer-term PYY-based therapies has been impeded by a lack of efficacy and side effects of nausea and vomiting (210, 211). The increase in PYY and GLP-1 secretion is thought to be a major factor in the sustained weight loss observed after gastric bypass surgery (166, 167, 212).

PPY is predominantly secreted by PP (F) cells in pancreatic islets adjacent to the duodenum, and like PYY, is responsive to food intake (195, 213, 214). Unlike PYY, however, its actions are mediated predominantly by Y4 receptors, coded for by the *NPY4R* gene on chromosome 10q11.22, which is expressed in the hypothalamus, coronary arteries, and ileum (215). Peripheral administration of PPY also causes a reduction in appetite and food intake in humans and mice, although interestingly intracerebroventricular injections in mice caused the opposite effect (216, 217). Transgenic mice overexpressing *Ppy* show a reduction in food intake and delayed gastric emptying (218). This effect appears to be mediated through Y4 receptors via an upregulation of *Bdnf* expression in the VMN, with concurrent downregulation of orexin (*Hcrt*) expression in the LHA (219). *Npy4r^−/−^* knockout mice exhibit weight gain and increased white adipose tissue accumulation (220).

#### Nucleobindin-2/nesfatin-1

Nesfatin-1 is the only known biologically active product of post-translational proteolytic cleavage of the 396 amino acid nucleobindin-2 (NUCB2) protein (encoded by *NUCB2* on chromosome 11p15.1) by PC enzymes to produce an 82 amino acid fragment (221, 222). In the hypothalamus, nesfatin-1 is found in the ARC, PVN, SON, and LHA in rats (223). *NUCB2* is also expressed elsewhere in the CNS and peripherally in adipose tissue, ghrelin-secreting gastric mucosal cells, and pancreatic islet cells (224-226). In a series of elegant experiments, Oh et al (223) demonstrated that of the by-products of NUCB2 processing, only nesfatin-1 was able to reduce food intake and weight gain in rats, the effects of which were antagonized by nesfatin-1 antibodies and NUCB2 antisense morpholino oligonucleotides. Importantly, this effect persisted even in leptin receptor-deficient rats but was abolished by pretreatment with SHU9119, an MC4R antagonist, indicating that the pathways through which nesfatin-1 operates are leptin-independent but POMC dependent. Nesfatin-1 positive neurons in the PVN and SON have been found to co-express OXT and arginine–vasopressin (AVP), and studies have suggested that nesfatin-1-induced oxytocinergic signaling in the PVN to POMC neurons in the NTS (but not the ARC) may be responsible for causing anorexia in the absence of the actions of leptin, with its effects blocked by an OXT antagonist (227-229). The discovery of nesfatin-1 has therefore helped elucidate the link between the appetite-regulating centers in the hypothalamus and brainstem. Despite this, however, the receptor for nesfatin-1 has yet to be fully characterized (230).

#### Oxytocin

OXT is a nonapeptide which is encoded by the *OXT* gene on chromosome 20p13, lying adjacent to the *AVP* gene but transcribed in opposite directions (231). The exomic sequence also encodes the carrier protein neurophysin I, such that OXT is synthesized as preproOXT-protein neurophysin I, which is then proteolytically cleaved by PC1/3 and PC2 during axonal transport from the hypothalamus to the posterior pituitary where it is stored until secretion, whereupon the free OXT peptide dissociates (232-238). OXT is predominantly secreted in the magnocellular neurons of the hypothalamic SON, and the magnocellular and parvocellular neurons of the PVN, with magnocellular projections to the posterior pituitary (239, 240). However, PVN neurons have also been demonstrated to send projections widely across the CNS (240-244). Conversely, neurons expressing *OXT* have also been found outside the hypothalamus indicating that OXT synthesis is not restricted centrally (234, 240, 245-251).

Although OXT has long held traditional roles in human parturition, lactation and ejaculation, its widespread expression and neuronal projections allude to its more recently described roles in a wider range of physiological and neurobehavioral processes. All of its functions are executed via a single G protein-coupled receptor encoded by the *OXTR* gene on chromosome 3p25.3 (252-254), which is expressed in numerous tissues apart from the breast, ovaries, endometrium, and myometrium. OXTR has been found throughout the brain (hypothalamus, basal ganglia, lateral septal nucleus, basal nucleus of Meynert, substantia nigra, NTS, substantia gelatinosa, and hypoglossal nucleus), adipose tissue, the kidneys, blood vessels, thymus, pancreas, adrenal glands, and osteoblasts (234, 253, 255).

Much of the discovery of the role of OXT in appetite and weight regulation has stemmed from animal experiments. Arletti et al (256). demonstrated that both central (intracerebroventricular) and peripheral (intraperitoneal) administration of OXT resulted in an anorexigenic effect in rats, with these effects being cancelled out by an OXT antagonist. It appears that 1 major site of action for OXT is the VMN (257), but OXT can also cause suppression of food intake by acting on the nucleus accumbens, part of the brain's reward circuitry (258, 259). Intraperitoneal administration of OXT in rats has been shown to cause widespread increased c-*fos* expression in the PVN, ARC, locus coeruleus, NTS, dorsal nucleus of the vagus, and area postrema, all of which are known to mediate energy homeostasis (260). The influence of OXT on appetite is carbohydrate-specific, and does not influence fat intake (261). *Oxt*^−/−^ knockout mice demonstrate a sustained preference for sweet tasting and carbohydrate-containing solutions over that of lipid-containing emulsions (262-265), while both *Oxt*^−/−^ and *Oxtr*^−/−^ knockout mice demonstrate long-term weight gain and increased white and brown adipose tissue deposition, but with reduced adrenaline production and aberrant cold-induced thermogenesis despite no significant differences in food intake (266-272). These findings support the hypothesis that OXT has a greater role in determining energy expenditure rather than suppressing appetite.

In humans, no known pathogenic human mutations in *OXT* or *OXTR* have been reported, and therefore the phenotypes of the human analogue of the *Oxt*^−/−^ and *Oxtr*^−/−^ knockout mice have never been described. Due to difficulties in measuring CNS and plasma OXT concentrations, studies on plasma OXT concentrations in relation to human obesity have produced mixed results, with some studies showing that peripheral concentrations are higher in obese subjects (273-276), while others demonstrate the opposite (277-280). Overall, however, the effect of OXT on appetite and weight is likely to be anorexigenic. More recently, the widespread use of the intranasal route for OXT administration has led to several studies analyzing its effects on appetite and weight. Intranasal OXT has been shown to reduce food intake and BMI in men with common obesity, while in healthy men, it reduced caloric intake and postmeal snack consumption with no change in appetite or energy expenditure (281-284). Disappointingly, in patients with defined HyOb syndromes, the effect of intranasal OXT has, however, been underwhelming. In Prader-Willi syndrome, intranasal OXT was associated with a reduction in food-related behavior but with no significant reduction in food intake, weight or BMI (285-287). Similarly, intranasal OXT demonstrated no effect on appetite in a craniopharyngioma survivor (288). Additionally, data on the correlation between measured plasma and cerebrospinal fluid OXT concentrations remains conflicting (289-295) and the pharmacokinetics of intranasal OXT is unclear (283, 285, 296-299). There are also marked difficulties in the measurement of OXT in biological fluids, particularly plasma, due to its inherent instability (300), low molecular weight, and low concentrations, making it subject to interference from other plasma proteins (301-304).

### Orexigens

#### Ghrelin

The discovery of ghrelin is unusual in that it was identified after its receptor, the growth hormone secretagogue receptor (GHSR), through a process of purification of various rat tissue extracts to identify fractions capable of activating it. Using this method, a 117 amino acid (prepro-ghrelin) encoded by the *GHRL* gene on chromosome 3p25.3 was identified, which then undergoes proteolytic cleavage by PC1/3 enzymes to produce ghrelin (28 amino acids), which is predominantly secreted in the oxyntic glands of the stomach (22, 305). Other sources of ghrelin secretion include the duodenum, jejunum and the lung, with smaller amounts found widely spread across various tissues (306, 307). Ghrelin is additionally activated through a process of acylation by ghrelin-O-acyltransferase (GOAT), which adds an O-n-octanoyl chain to serine-3 of the peptide molecule (308). Acylation is a necessary step for ghrelin to be able to activate GHSR, and is postulated to be how the function of ghrelin is regulated (22, 309). GHSR itself is expressed in the rat and human hypothalamus (particularly the ARC), pituitary, hippocampus, and area postrema, with small amounts in the pancreas (310, 311).

Stimulation of GHSR by acylated ghrelin induces GH release independent of, and more potently, than GHRH (312). Both dominant and recessive mutations in *GHSR* have been described in humans in association with short stature, some of whom had classical GH deficiency (313, 314). The role of ghrelin in modulating appetite and weight was first described by Tschop et al (315), where both subcutaneous and intracerebroventricular administration of ghrelin in mice led to weight gain (with the latter also causing hyperphagia), independent of its effects on GH secretion and the NPY signaling pathway. The effects of ghrelin on weight were largely mediated by a reduction in fat catabolism. Ghrelin concentrations were also increased by fasting and decreased by re-feeding, particularly after an oral glucose load (315). Other rodent studies have however suggested that ghrelin lies upstream of the NPY/AgRP and POMC/CART pathway, with ghrelin-induced feeding being inhibited by antagonists and antibodies of NPY and AgRP, αMSH, and leptin (316). Interestingly, *Ghrl^−/−^* knockout mice display no abnormalities and have similar responses to fasting and overfeeding as their wild-type littermates (317). Contrastingly, *Ghsr^−/−^* knockout mice show a reduction in weight in the long-term, particularly in response to a high-fat diet where food intake was also impaired (318, 319). *Mboat4^−/−^* (the gene for GOAT) knockout mice do not show any phenotypic differences in response to a standard or high-fat diet, but long-term caloric restriction leads to increased weight loss and hypoglycemia due to GH insufficiency (320).

In humans, plasma ghrelin is negatively correlated with BMI and fat mass (321), and is increased after fasting and in anorexia nervosa (306). Total plasma ghrelin concentrations are also increased in Prader–Willi syndrome, the archetypal genetic HyOb disorder, compared with BMI-matched controls even in young children, regardless of growth hormone (GH) treatment and preceding the onset of obesity (322-324). In this disorder, hyperphagia and weight gain is not apparent at birth and only develops in the second “nutritional phase,” corresponding to an increase in the acylated: unacylated ghrelin ratio (325, 326). Administration of exogenous ghrelin has been shown to increase the subjective sensation of hunger and objective food intake in obese and lean human subjects (327, 328). However, to date, there have been no successful trials of a ghrelin antagonist leading to weight loss in humans.

#### The agouti-related peptide/neuropeptide Y system

The AgRP/NPY system is closely related anatomically to the POMC/CART system with both sets of neurons being located in the hypothalamic ARC, but with their appetite-regulating peptide expression patterns being mutually exclusive and working in opposing directions (329, 330). AgRP is a 112 amino acid peptide related to agouti, a mouse protein which, when mutated in a heterozygous state, causes yellow fur (due to MC1R antagonism) and obesity (due to MC4R antagonism) (331, 332). AgRP is encoded by the *AGRP* gene on chromosome 16q22.1 and expression in mice and humans is restricted to the adrenal cortex and medulla, hypothalamus (ARC and median eminence), subthalamic nucleus and testis, with weaker signals in the lungs and kidney. ARC expression is increased in *ob*/*ob* (leptin deficient) and *db*/*db* (leptin receptor deficient) mice, suggesting that AgRP lies downstream of the leptin-dependent appetite-regulating pathway (21). Indeed, *FoxO1* mutations in rodents lead to the loss of the ability of leptin to suppress *AgRP* expression, demonstrating the role of the PI3K/PKB/FOXO1 pathway in linking leptin with AgRP secretion (63). Subsequent experiments by Ollmann et al (333). and Graham et al (334) demonstrated that AgRP is a selective MC3R and MC4R competitive antagonist inhibiting the effects of αMSH, with transgenic mice overexpressing the human *AGRP* and mouse *Agrp* genes gaining significant amounts of weight and developing glucose intolerance and insulin insensitivity compared with their littermates, but with no change in pigmentation (ie, no effects on MC1R). Conversely, ablation of NPY/AgRP neurons leads to starvation (335). Expression is increased by fasting in rodent models, and plasma concentrations are positively correlated with BMI in humans (330, 336). Intracerebroventricular infusions of AGRP in mice generate a potent and prolonged increase in appetite, activating pathways involving the NTS, LHA, amygdala and nucleus accumbens, while antagonizing the effects of melanotan-II (an MC4R agonist) and αMSH (337-339).

The vast majority of AgRP neurons also coexpress NPY, the final known member of the family of appetite-regulating neuropeptides (330). Like the other members with which it shares sequence homologies, PYY and PPY, NPY is a 36 amino acid peptide, encoded by the *NPY* gene on chromosome 7p15.3 (340, 341). Similar to PYY, NPY is proteolytically cleaved by DPP4 to form NPY_3-36_, rendering it less effective at activating the Y1 receptor (200, 342). Apart from being colocalized in AgRP neurons in the hypothalamic ARC as detailed above, *NPY* expression is widespread within the nervous system, and has been demonstrated in the neuronal plexi of the small and large intestines, the sympathetic neurons of the autonomic nervous system, the spinal cord, SON, PVN, DMN, NTS, medulla, locus coeruleus, hippocampus, amygdala, basal ganglia, nucleus accumbens, and cerebral cortex (192, 330, 343-350). Additionally, NPY has been found in adipose tissue (particularly visceral adipocytes), adrenal medulla, blood vessels, and activated lymphocytes and monocytes (351-354).

Like AgRP, NPY expression is increased in the fasted state as well as in the *ob/ob* mouse, indicating that it, too, lies downstream of anorexigens such as leptin (354-357). Indeed, administration of insulin and leptin both result in a reduction of NPY expression in the ARC, while ghrelin has the opposite effect (77, 316, 358). Correction of leptin deficiency in the *ob/ob* mouse similarly reduces *Npy* expression (359). *ob/ob Npy^−/−^* double knockout mice have reduced hyperphagia and weight gain, an increased energy expenditure and a greater degree of physical activity compared to *ob/ob* mice (360). However, *Npy^−/−^* mice are still able to decrease their food intake and lose weight when treated with leptin, indicating that reduction in NPY is additive to, but not essential for leptin's anorexigenic effects (361). Similarly, NPY is increased in the ARC, PVN, VMN and LHA in T1DM, the effect of which is reduced by insulin (362, 363).

Unlike PYY and PPY, the actions of NPY are mediated via the full range of Y1, Y2, Y3, Y4, and Y5 receptors (344, 350). In the gut, Y1 and Y2 receptors mediate NPY's effects on inhibiting gastrointestinal motility and secretion (364-367). However, the major effect of NPY on the CNS is orexigenic and mediated through central Y1 and Y5 receptors, with rats receiving intracerebroventricular NPY demonstrating increased food intake (mainly via the PVN, by increasing portion sizes and carbohydrate intake), white adipose tissue accumulation and weight gain, which was largely mediated by the hyperphagia observed rather than a reduction in energy expenditure (368-371).

#### Hypocretin/orexin

The hypocretin (*HCRT*) gene on chromosome 17q21.2 encodes 2 peptides, hypocretin 1 (orexin A, 33 amino acids) and hypocretin 2 (orexin B, 28 amino acids) which are the result of proteolytic cleavage of preprohypocretin (372, 373). The effects of the hypocretins are mediated via their receptors, hypocretin receptors 1 (HCRTR1) and 2 (HRCTR2), with HCRTR1 being selective for hypocretin 1 and HRCTR2 being nonselective (373). *Hcrt* expression has been found in the LHA (particularly the perifornical area), DMN, and ventral thalamus, with neurons projecting widely, including to the PVN, locus coeruleus, and NTS (372-375). The widespread connections of these neurons allude to the variety of pathways involving the hypocretin system, the most well-known being its involvement in regulating arousal and sleep–wake cycle behaviors (376, 377). *Hcrt^−/−^* mice exhibit sleep dysregulation, with sleep-onset rapid eye movement and more fragmented non-rapid eye movement sleep (378). However, only a single case of a heterozygous *HCRT* mutation associated with the narcolepsy–cataplexy syndrome has been described in humans thus far (379). Otherwise, this disorder has been associated with human leukocyte antigen subtypes, supporting an immune-mediated rather than genetic pathogenesis (380).

One of the early clues supporting the role of the hypocretins in appetite and weight regulation was the late-onset obesity observed in mice which had undergone genetic ablation of *Hcrt*, despite a reduction in food intake compared with their wild-type counterparts (381). Patients with narcolepsy also have an increased tendency to being overweight or obese (382). The perifornical area of the LHA has been shown to be a major site of NPY-induced feeding, where NPY axons synapse with neurons containing hypocretin (329, 383). Additionally, the locus coeruleus is densely innervated by hypocretin-secreting neurons, which increase its noradrenergic output as well as arousal and locomotor activity in rats (374). Pharmacogenetic activation of *Hcrt* neurons increases food intake but also locomotor activity and energy expenditure (384). Additionally, both hypocretins are orexigenic when administered intracerebroventricularly in rats (373). Similarly, *Hcrt* expression is upregulated by fasting and reduced by insulin (373, 385). In humans, plasma hypocretin 1 is negatively associated with BMI (386).

However, the overall effect of the hypocretin system on appetite is far from straightforward, with other experiments demonstrating varying and occasionally opposing effects. Increased *Hcrt* signaling results in resistance to high-fat diet–induced obesity in the presence of a reduced food intake and increased energy expenditure, an effect largely mediated via *Hcrtr2* (387, 388). The same mice involved in these experiments were also resistant to age-related weight gain, an effect thought to be due to the loss of hypocretin neurons over time in animal models (377, 389). Some authors have suggested that these differences in the effects of hypocretins may be related to the environment they are studied in, as other signaling molecules secreted by *Hcrt* neurons may also be impaired in experiments involving neuronal destruction rather than those using more targeted genetic knockout techniques (390).

### Other Appetite-Regulating Peptides

Several other peptides have been described in the literature, particularly in animal models, to be involved in appetite regulation. However, their role in humans has yet to be fully elucidated, and to date no clear human phenotypes of equivalent animal models have been described. Amongst these are melanin-concentrating hormone (MCH; gene *PMCH* on chromosome 12q23.2, 19 amino acids), which has been shown in rodent models to be present in neurons in the LHA alongside *Hcrt*-expressing cells, acting on MCH receptors (encoded by *MCHR1* and *MCHR2*) throughout the nervous system (329, 391, 392). Evidence from rodent knockout models of *Promch* and *Mchr1*, as well as overexpressing *Promch* transgenic mice, suggest that MCH acts as an orexigen, stimulating feeding and weight gain (393). To date, however, only genetic variants of unknown significance and single nucleotide polymorphisms have been identified in *PMCH*, *MCHR1*, and *MCHR2* to be associated with obesity in humans, although 2 inactivating *MCHR1* mutations have been identified in underweight individuals (393, 394).

Similarly, adiponectin (gene *ADIPOQ* on chromosome 3q27.3, 244 amino acids) is produced by adipocytes (395). Unlike leptin, however, plasma adiponectin is negatively correlated with BMI, and is increased after bariatric surgery (396, 397). Despite this, adiponectin (*Acrp30*) knockout mice do not demonstrate differences in weight compared to their wild-type counterparts, and animal models suggest that the role of adiponectin is largely in the mediation of insulin sensitivity, lipid clearance, vascular remodelling, and inflammation (398, 399). The effects of intracerebroventricular adiponectin on appetite and weight are conflicting (400, 401), and to date, no human phenotypes involving disordered energy homeostasis and adiponectin mutations have been described.

Other peptides such as CRH, GHRH, TRH, somatostatin (SS), resistin, interleukin-1β, and 5-HT have also been described in the literature as having appetite- and/or weight-regulating effects but will not be discussed here (40, 41, 329, 402-404). Additionally, the role of inflammation in obesity is only beginning to be characterized, with the involvement of peptides such as GDF15 only starting to be elucidated (405).

### Current Understanding of Appetite and Weight Homeostasis in Man

**Figure 1.**
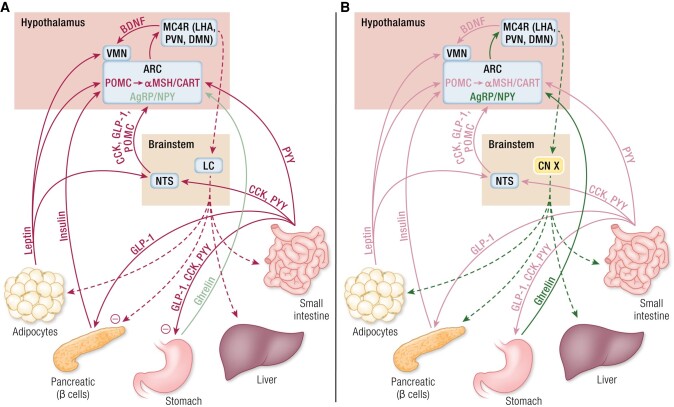
Schematic illustrating the complexity of currently elucidated major anorexigenic and orexigenic appetite- and weight-regulating circuitry. Solid arrows signify afferent pathways, dashed arrows signify efferent pathways. (A) In the fed state, peripheral anorexigenic pathways signal via the NTS and directly to the VMN/ARC to ultimately increase αMSH/CART agonism on MC4R and reduce appetite. This then signals via efferent pathways, largely via the sympathetic nervous system to increase various compensatory catabolic pathways, including glycogenolysis, lipolysis, thermogenesis and suppression of insulin secretion. (B) In the fasted state, the anorexigenic “brakes” are released, leaving the main peripheral orexigen, ghrelin to increase AgRP/NPY antagonism of MC4R and increase appetite. Efferent pathways, largely acting via the vagus nerve, then increase various compensatory anabolic pathways, including lipogenesis, peristalsis, and postprandial insulin secretion, partly via GLP-1. αMSH, α-melanocyte-stimulating hormone; AgRP, agouti-related peptide; ARC, arcuate nucleus; BDNF, brain-derived neurotrophic factor; CART, cocaine- and amphetamine-regulated transcript; CCK, cholecystokinin; CN X, vagus nerve; DMN, dorsomedial nucleus; GLP-1, glucagon-like peptide 1; LC, locus coeruleus; LHA, lateral hypothalamic area; MC4R, melanocortin receptor 4; NPY, neuropeptide Y; NTS, nucleus tractus solitarious; POMC, proopiomelanocortin; PVN, paraventricular nucleus; PYY, peptide YY; VMN, ventromedial nucleus.

The regulation of appetite and weight in humans is therefore governed by a balance between anorexigenic and orexigenic signaling pathways, with obesity being the result of an imbalance between energy intake and expenditure. In the fed state (Fig. 1A), there is an increase in leptin secretion from adipocytes, activating its receptors in the hypothalamus, particularly in the VMN and ARC, to signal an increase in adiposity (48, 56). Concurrently, insulin is released by pancreatic β-cells in response to an increase in blood glucose concentration. Both leptin and insulin act in concert via the PI3K/PKB/FOXO1 signaling pathway to stimulate POMC/CART and inhibit AgRP/NPY neurons in the ARC, with leptin additionally increasing POMC synthesis via the JAK/STAT pathway (59-64, 76, 77, 79).

Simultaneously, other peripheral peptides are secreted along the gastrointestinal tract in response to feeding. GLP-1 is secreted by enteroendocrine L cells in response to the presence of glucose, potentiating insulin secretion, delaying gastric emptying, and suppressing orexigenic pathways in the hypothalamus and NTS (145, 146, 149, 161, 163). CCK, produced by the small intestine, acts on CCKAR in the stomach (where it delays gastric emptying) and the NTS, the latter sending its own CCK- and POMC-mediated signals to the hypothalamus to promote anorexigenic signaling via MC4R (96, 175, 182). PYY and PPY, secreted by enteroendocrine L cells and pancreatic PP cells respectively, both cause delayed gastric emptying, with PYY activating POMC and inhibiting NPY neurons, while PPY increases BDNF and reduces HCRT synthesis, thereby inducing an overall anorexigenic effect (205, 219).

Centrally, activation of POMC/CART neurons in the ARC and NTS, and the subsequent generation of αMSH to act on MC3R and MC4R in the ARC, PVN, DMN and LHA of the hypothalamus, as well as CART, leads to a suppression of food intake and an increase in energy expenditure (97, 103, 105, 118, 119, 329). Concurrently, while the role of BDNF in promoting anorexia is still being fully elucidated, it appears that its signaling is both stimulated by and responsible for promoting POMC secretion, while also increasing energy expenditure through increased physical activity (131, 135, 137). Additionally, the orexigenic AgRP/NPY pathway is suppressed by leptin and insulin, and by the lack of the main peripheral orexigenic hormone, ghrelin (77, 316, 358).

Increased central anorexigenic signaling then increases output through its efferent arm, which is largely mediated by the sympathetic nervous system via the locus coeruleus (406-408). The sympathetic nervous system is then responsible, via β-adrenoceptors, for increasing various catabolic processes such as mitochondrial biogenesis, glycogenolysis, lipolysis, thermogenesis, and increased activity, supported by an increase in TRH signaling (1, 42), Concurrently, insulin secretion is suppressed via α-adrenoceptors, thus closing the feedback loop (409). The PVN is also responsible for secreting TRH, CRH, and OXT, all of which are able to suppress food intake and/or increase energy expenditure (40, 118, 119, 329, 402, 410, 411).

Conversely, in the fasted state (Fig. 1B), leptin and insulin signaling is suppressed alongside other peripheral anorexigens (due to a lack of calories within the gastrointestinal tract), therefore removing the “brakes” on the appetite-regulating circuitry (48). Additionally, gastric ghrelin secretion is increased (316). A consequent reduction in POMC/CART signaling leaves the orexigenic AgRP/NPY pathway unopposed, MC3R and MC4R occupancy free to be antagonized by AgRP, and for NPY secretion to increase (63, 333, 334, 355-357). The lack of opposition to the orexigenic drive also increases the effects of the hypocretins, particularly in the LHA, which are also stimulated by fasting (373, 385). The overall effect is therefore one of a reduction in sympathetic nervous system activity, and, via the PVN and LHA, an increase in vagal nerve firing via projections to the medial longitudinal fasciculus (412). This subsequently reduces energy expenditure and increases various anabolic processes, including lipogenesis (eventually increasing leptin secretion), peristalsis and nutrient absorption, and postprandial insulin secretion, thereby closing the feedback loop (1, 413-415). Part of this latter response is additionally mediated by GLP-1, which in itself is an anorexigen.

## The Pathophysiology of Hypothalamic Obesity

The complexity and redundancy of the various hypothalamic-gut-brainstem appetite-regulating pathways has therefore made elucidating the “final common pathway” that could help identify novel targets for the treatment of both obesity and HyOb difficult. To date, only monogenic HyOb disorders resulting in deficiencies of anorexigens have been described in humans; the lack of equivalent mutations resulting in overexpression of orexigens has meant that this arm of appetite regulation is less well understood. Additionally, limits in the resolution of current magnetic resonance imaging techniques has meant that the neuroanatomical and functional study of the hypothalamus and its widely projecting connections is difficult, not just in healthy subjects, but even more so in diseased states where its normal neuroanatomical structure can be distorted. Patients with HyOb have classically been described as hyperphagic, and this is supported by the phenotype demonstrated in the majority of the monogenic obesity syndromes involving mutations of genes participating in the leptin/POMC/CART/MCR signaling pathway.

Studies of the pathophysiology and management of HyOb were initially largely dominated by 2 major hypotheses—firstly, that hypothalamic (VMN) damage leads to impaired satiety, hyperphagia, obesity and eventual hyperinsulinemia (ie, predominantly afferent pathway driven); and secondly, that VMN damage leads to disinhibited vagal innervation of pancreatic β-cells, hyperinsulinemia, and obesity. After the early work on the roles of different hypothalamic centers in energy homeostasis by Smith (9) and Hetherington and Ranson (10), studies in the 1980s began to recognize the role of the efferent pathway, stemming from studies (416) which demonstrated that hyperphagia, obesity, and hyperinsulinemia was attenuated in streptozotocin-induced diabetic rats with VMN lesions who additionally had fetal pancreatic tissue transplanted under their renal capsules, indicating that an intact vagus nerve was necessary for their development. Subsequent experiments in rats with VMN lesions showed that subdiaphragmatic vagotomy reversed the HyOb and hyperinsulinemia usually observed, primarily by reducing food intake (417). These studies then led to attempts to curb hyperinsulinemia in HyOb, but with only marginal success at weight loss, indicating that the lipogenic and anabolic effects of insulin were not the sole drivers of weight gain.

In humans, several lines of evidence suggest that the role of a reduction in energy expenditure cannot be underestimated as a predominant mechanism resulting in weight gain. This hypothesis is supported by studies of Prader–Willi syndrome patients, the archetypal syndromic form of HyOb. In this disorder, infants present with feeding difficulties from birth (phase 1a) but severe hyperphagia in later life (phases 3-4) (325). Prior to the onset of hyperphagia in early to midchildhood (phase 2b), however, weight gain is observed with no observable abnormalities in appetite and a normal caloric intake for age (phase 2a). This is associated with a concomitant reduction in resting energy expenditure (REE).

In studies of acquired causes of HyOb, pediatric patients with craniopharyngiomas have been shown to consume less calories than BMI-matched controls with common obesity, mainly by a reduction in fat intake (418). Concurrently, through accelerometry, the same authors demonstrated that the amount of physical activity undertaken by HyOb patients was reduced compared with their counterparts with common obesity. While it could equally be postulated that food intake diaries are inaccurate at measuring true caloric intake and instead reflect the perception of participants, in this pediatric study, it would have been hard not to imagine that at least a proportion of these diaries had been completed by the parents of the participants rather than the participants themselves. Additionally, ad libitum food intake studies, widely regarded as the gold standard for studying appetite and satiety, are difficult to ethically perform in the pediatric setting (419). Similarly, another study of pediatric patients with HyOb due to various causes also demonstrated that REE in these patients was significantly reduced compared to participants with common obesity (420). These authors however found that there were no significant differences in caloric intake between the groups.

The mechanisms underlying this reduction of energy expenditure largely remain to be elucidated. While the idea that a decrease in sympathetic nervous system output via efferent appetite-regulating pathways would seem obvious, the evidence supporting this is conflicting. In 1 study comparing plasma catecholamine responses to insulin-induced hypoglycemia in children with craniopharyngioma to short normal age-matched controls, the peak plasma adrenaline was lower, while the peak plasma noradrenaline was higher in the former group, with a lower 24-hour urinary excretion of adrenaline, dopamine and vanillylmandelic acid (VMA) (421). A similar study of adult craniopharyngioma patients had previously reported similar findings (422). It is worth noting, however, that the median and mean BMIs of craniopharyngioma participants in this latter study were 0.5 (interquartile range 0.1-1.8) SDS and 28.3 ± 5.1 kg/m^2^, respectively (ie, not all obese). Similarly, a study comparing spot urinary catecholamines in survivors of pediatric-onset craniopharyngiomas demonstrated that urinary homovanillic acid and VMA concentrations were significantly lower in obese patients with craniopharyngioma than those with a normal BMI (who had comparable values to controls), and this was related to hypothalamic involvement of the tumor rather than irradiation or degree of tumor resection (423). However, a further study of adolescents and young adults with craniopharyngiomas and HyOb showed that heart rate variability (a measure of autonomic nervous system activity) and 24-hour urinary adrenaline (epinephrine), noradrenaline (norepinephrine), metanephrine, normetanephrine, and VMA output was not significantly different to that of age-, sex-, and BMI-matched controls, despite a lower REE and increased Epworth sleepiness scale scores (424, 425).

Taken together, these data suggest that a possible mechanism (particularly in acquired HyOb which has been studied in more detail) is that an early reduction in sympathetic nervous system output, and consequently a reduction in REE, leads to weight accumulation and eventual HyOb over time in patients at risk. Comparatively, it also suggests that once common obesity is attained through a chronic increase in caloric intake, the biochemical and endocrine processes observed are virtually identical to that of HyOb.

### Monogenic “Nonsyndromic” Hypothalamic Obesity Syndromes

As discussed above, the advent of the molecular genetic era has led not just to the discovery of a wide range of appetite- and weight-regulating peptides involved in the hypothalamic–gut–adipose tissue neuroendocrine circuitry controlling energy homeostasis, but also their associated monogenic HyOb syndromes involving mutations in their genes (Table 1). All of these syndromes are associated with hyperphagia, particularly those involving the leptin—POMC/CART—MC4R and BDNF—TRKB pathways. Replacement therapy of some of these deficient peptides, or their downstream effectors, has already proven successful in some of these disorders.

**Table 1.** Monogenic nonsyndromic causes of hypothalamic obesity and current evidence-based targeted treatments in humans

Protein (gene)	Inheritance	Primary mechanism	Other phenotypic features	Evidence-based targeted treatments for human obesity
Leptin (*LEP*) (14, 426)	Autosomal recessive	Leptin deficiency	Hypogonadotropic hypogonadism, immune system dysfunction (CD4+ T cell lymphopenia), postural hypotension	Recombinant leptin (15)
Leptin receptor (*LEPR*) (45)	Autosomal recessive	Leptin resistance	GH deficiency, central hypothyroidism, hypogonadotropic hypogonadism	Setmelanotide (MC4R agonist) (427)
Proopiomelanocortin (*POMC*) (428, 429)	Autosomal recessive	POMC (αMSH precursor) deficiency	ACTH deficiency, red hair, pale skin (GH deficiency, central hypothyroidism, hypogonadotropic hypogonadism)	Setmelanotide (430)
Melanocortin 3 receptor (*MC3R*) (431)	De novo heterozygous	αMSH resistance	None described	None
Melanocortin 4 receptor (*MC4R*) (106, 107)	Autosomal dominant	αMSH resistance	None described	Setmelanotide (432)
Cocaine- and amphetamine-regulated transcript (*CARTPT*) (124)	Autosomal dominant	CART deficiency	None described	None
Brain-derived neurotrophic factor (*BDNF*) (137, 138)	De novo heterozygous	BDNF deficiency	Cognitive impairment, hyperactivity; or as part of the 11p13-14 contiguous gene deletion WAGRO syndrome (Wilms tumor, aniridia, genitourinary abnormalities, mental retardation, obesity)	None
Tyrosine receptor kinase B (*NTRK2*) (141)	De novo heterozygous	BDNF resistance	Global developmental delay, short-term memory impairment, behavioral stereotypies, impaired nociception	None
Prohormone convertase 1/3 (*PCSK1*) (429, 433, 434)	Autosomal recessive	Failure of cleavage of proinsulin to insulin, proglucagon to glucagon/GLP-1/GLP-2, pro-CCK to CCK-8, POMC to α-MSH/ACTH, pro-CART to CART, proghrelin to ghrelin, pro-AgRP to AgRP, pro-GHRH to GHRH, pro-GnRH to GnRH, pro-AVP to AVP	GH, LH, FSH, ACTH, AVP deficiencies, impaired glucose tolerance/diabetes mellitus, postprandial hypoglycemia, malabsorptive diarrhea	None
Single-minded homolog 1 (*SIM1*) (435-437)	De novo heterozygous	Disrupted hypothalamic development (particularly of the SON and PVN, with reduced MC4R expression and OXT, AVP, TRH, CRH and SS neurons)	Hypogonadotropic hypogonadism, facial dysmorphism, behavioral difficulties, “Prader–Willi syndrome-like” phenotype	None
SH2B adaptor protein 1 (*SH2B1*) (438)	Autosomal dominant/de novo heterozygous)	Disrupted leptin and insulin signaling (via JAK/STAT pathway)	As part of the 16p11.2 contiguous gene deletion syndrome, mild developmental delay	None
Steroid receptor coactivator-1 (*SRC1*) (439)	De novo heterozygous	Disrupted leptin signaling (via impairment of STAT3 pathway which stimulates *POMC* transcription)	None described	None

Although these forms of HyOb are often classified as “nonsyndromic” disorders, additional phenotypic features are often observed, alluding to the interconnectivity of these pathways with other aspects of neuroendocrine function. For instance, congenital leptin deficiency and leptin receptor mutations both result additionally in hypogonadotropic hypogonadism (in contrast to common obesity where puberty is often early (14, 15, 45, 426). This is because leptin acts as a “metabolic gate” signaling the fed state, with a permissive effect on the onset of puberty by modulating GnRH secretion (440-442). Recombinant human leptin replacement therapy has been found to reverse this clinical finding (15). Similarly POMC deficiency can result in a clinical phenotype not just easily attributable to deficits in the secretion of all its constituent peptides (HyOb [αMSH], central hypoadrenalism [ACTH], red hair, and pale skin [αMSH]), but also consisting of other hypothalamo-pituitary hormone deficiencies due to interactions between the POMC system and these axes (428, 443). Mutations in the BDNF/TRKB pathway, due to its role in supporting neuronal survival, are predictably associated with neurocognitive defects (137, 141). Finally, mutations in genes affecting hypothalamic development (*SIM1*), post-translational processing of appetite-regulating hormones (*PCSK1*), or common downstream signaling pathways (*SH2B1*) lead to HyOb through their impact on multiple appetite- and weight-regulating hormones (429, 435, 438).

### Syndromic Forms of Obesity Without Hypothalamic Structural Defects

Syndromic obesity is defined as the presence of obesity along with characteristic additional pleiotropic clinical features such as developmental delay, dysmorphisms, and other congenital anomalies. In comparison with monogenic HyOb syndromes with “nonsyndromic” additional clinical features, the biological link between these disorders and obesity largely remains unknown, or is not attributable to a single gene defect. While only a handful of monogenic HyOb syndromes have been identified, a recent systematic review identified 79 unique obesity syndromes in the literature, of which only 19 had been fully genetically elucidated (444). However, it is worth noting that with the current advancements in molecular genetics, over time, the genetic pathways linking some of these disorders with obesity may be discovered, leading to their reclassification as a monogenic HyOb syndrome. While it is beyond the scope of this chapter to discuss all of these disorders, the commonest genetic form of syndromic HyOb, Prader–Willi syndrome, will be reviewed here.

#### Prader–Willi syndrome

Prader–Willi syndrome is, in effect, a contiguous gene deletion syndrome of the paternal copies of several imprinted genes in the 15q11-13 region (*SNRPN*, *NDN*, *MAGEL2*, *MKRN3*, *SNORD116*), with a birth incidence of 1 in 20 000 and an overall population prevalence of 1 in 52 000 (445). The deletion of the paternally expressed genes can either arise directly (75%), or through maternal uniparental disomy (22%), imprinting errors (3%) or paternal chromosomal translocation (<1%) (446, 447). The major diagnostic criteria include neonatal and infantile hypotonia, feeding difficulties with a poor suck and poor weight gain in infancy (usually requiring nutritional support), followed by rapid-onset weight gain and hyperphagia in childhood, characteristic facial dysmorphisms, hypogonadism, developmental delay or learning difficulties, and 1 of the genetic defects described above (448). More recently, the nutritional phases observed have been described in greater detail. As detailed above, weight gain and a reduction in REE (phase 2a) precedes the hyperphagia observed in childhood (phase 2b) (325). Hypothalamo-pituitary dysfunction is a recognized component of this disorder, and apart from the severe HyOb and hyperphagia observed (leading to parents often needing to lock fridges and cupboards at night), deficiencies in GH, luteinizing hormone, follicle-stimulating hormone (FSH), and ACTH have been described (449, 450).

From an appetite regulation perspective, the most notable finding observed in Prader**–**Willi syndrome is an increase in circulating ghrelin preceding the onset of hyperphagia (322-324, 450, 451). Importantly, the plasma acylated to unacylated ghrelin ratio is increased, due to an overall increase in acylated ghrelin (326). Contrastingly, in common obesity, both forms of circulating plasma ghrelin were decreased as an expected compensatory response to a positive energy balance. The mechanism behind this difference remains unclear. Additionally, post-prandial PPY secretion is impaired (452, 453). The literature surrounding measurement of plasma OXT concentrations in Prader–Willi syndrome is conflicting, with some authors finding it is decreased relative to the degree of obesity, while others have reported that both plasma and cerebrospinal fluid OXT is increased (451, 454, 455). Confusingly, postmortem studies suggest that the number of OXT neurons in the PVN is reduced in these patients (456). To date, the only evidence-based endocrine therapy for this disorder is GH, which results in an improvement in body composition, REE, bone mineral density and muscle strength on top of linear growth (446, 457). Other treatment trials, including SS analogues, PPY and OXT have failed to yield long-term results in terms of a reduction in food intake or weight gain, although OXT may improve the feeding difficulties in infancy and behavioral difficulties in childhood and later life (285-287, 322, 458, 459).

### Hypothalamic Obesity in Congenital and Acquired Structural Hypothalamic Disorders

Unlike monogenic HyOb syndromes, the pathophysiology of HyOb in congenital (eg, SOD) and acquired (eg, suprasellar tumors, traumatic brain injury, hypophysitis) disorders of the hypothalamo-pituitary region is less clear. The size of the hypothalamus precludes detailed anatomical study using current magnetic resonance imaging techniques (in clinical practice this is usually 1.5-3T), although higher strength scanners (up to 7T) may help improve the visualization of this structure (460). However, to date, there are few data on the correlation between hypothalamic structure and function, particularly in mapping out its connectivity with other regions within the CNS (461-464). Moreover, when normal neuroanatomy is distorted by congenital malformations or disrupted by acquired diseases such as suprasellar tumors, interpretation becomes even more difficult. Lastly, it is likely that the hypothalamic injury represented by these disorders is widely heterogenous, and the degree of damage or disruption to the individual nuclei is highly variable. We discuss the commonest causes of congenital (SOD) and acquired (brain tumors) hypothalamic obesity here.

#### Septo-optic dysplasia

SOD is a rare developmental disorder of the forebrain, optic pathway and pituitary gland, with an incidence of about 1 in every 10 000 births (465). It is loosely defined by the presence of at least 2 of 3 components of the triad of optic nerve hypoplasia, midline forebrain defects (eg, agenesis of the corpus callosum, absent septum pellucidum) or hypopituitarism (27, 465). While SOD is a congenital malformation disorder, its etiology is multifactorial, with both genetic and environmental factors being implicated. For instance, the incidence of SOD is correlated with indices of deprivation such as unemployment and teenage pregnancies (465). Variants in a wide range of hypothalamo-pituitary developmental transcription factors have also been described in association with SOD (eg, *HESX1*, *OTX2*, *SOX2*, *PROKR2*, *KAL1*, *FGF8*, *TCF7L1*), but in the majority (99%), the underlying etiology is not identifiable (27, 466-470). Many of these genes also show variable expressivity, with an overlap with other disorders such as Kallmann syndrome (*KAL1*, *FGF8*, *FGFR1*) or combined pituitary hormone deficiency (*HESX1*, *PROKR2*) (471-474).

HyOb develops in 31% of patients with SOD, and can occur even in 12% of patients with isolated optic nerve hypoplasia (28). Associated neurological features are frequent (57% with bilateral optic nerve hypoplasia), ranging from focal neuro-ophthalmological deficits to developmental delay (475, 476). Additionally, social communication, and repetitive or restrictive behavioral difficulties can occur in a third of patients (477). The combination of visual impairment, behavioral and sleep difficulties, hyperphagia, and weight gain seen in this condition can often be extremely difficult to manage. Some of these features are likely caused by hypothalamic dysfunction, although this is difficult to demonstrate.

#### Central nervous system tumors

CNS tumors are the commonest cause of acquired HyOb and the second commonest childhood malignancy, accounting for 25% of cancers in children <15 years with an annual incidence of 35 cases/million/year, which is rising each year due to improvements in diagnosis (478-483). More than 80% of childhood CNS tumor survivors develop at least 1 endocrine deficit, GH deficiency being most frequent (484). The etiology of these endocrinopathies is multifactorial, particularly with suprasellar tumors which lie in close proximity to the hypothalamus and pituitary gland, accounting for 5% to 16% of all CNS tumors in childhood and young adulthood (485). In this scenario, hypothalamo-pituitary dysfunction and HyOb can be secondary to tumor- (location, histology) or treatment-related (neurosurgery, radiotherapy) factors (39). The risk of HyOb has been shown to be increased with the extent of neurosurgical resection, and therefore more conservative surgical approaches are increasingly being advocated (486-488). However, although much of HyOb has often been blamed on iatrogenic damage from surgical interventions or radiotherapy, 1 longitudinal study of craniopharyngiomas showed that increases in BMI SDS often occurred months to years preceding the diagnosis, with hypothalamic tumor involvement being a significant risk factor (489).

It is worth noting that 5-year survival for this subgroup of pediatric CNS tumors is high, particularly as the 2 commonest histologies found in this region are benign (craniopharyngiomas 95%, low-grade gliomas 96% (31, 490)). Despite this, HyOb is significantly over-represented in this subcohort and, like SOD, this is often coupled with visual deficits and neurobehavioral dysfunction (491-494), making management of these patients, who have already survived 1 life-threatening disorder, complex.

## Current Management Strategies for Hypothalamic Obesity

### Overview of Current Management Strategies for Common Obesity

The complexity of managing a rare disorder such as HyOb must be understood in the context of the difficulties in treating common obesity in childhood. To date, no single lifestyle or medical intervention has been identified which is able to produce sustained weight loss. Several systematic reviews have been conducted examining the efficacy of diet, physical activity and behavioral (lifestyle) interventions, drug treatments, and surgical procedures on obesity in children and young people (Table 2). In terms of lifestyle interventions, 3 meta-analyses of randomized controlled trials (RCTs) have been conducted, stratified by age, all of which show marginal reductions in BMI over relatively short periods of follow-up ranging from a mean difference in long-term BMI SDS reduction of −0.01 in 6- to 11-year-olds (*P* = .56) to −0.25 in those under 6 years (*P* = .0013) (495-497). The interventions examined were widely heterogeneous, and included dietary and lifestyle counseling, physical activity training programs, sponsored gym memberships, and behavioral therapy, targeted at individuals, families or groups. Interestingly, the effects were more sustained in the pre-school and adolescent age groups but none of these studies examined outcomes in adulthood.

**Table 2.** Summary of Cochrane meta-analyses of interventions for treating childhood obesity

Intervention	Number of RCTs	Number of participants, n	Mean difference in ΔBMI, kg/m^2^ (95% CI, n)	Mean difference in ΔBMI SDS (95% CI, n)	Duration of intervention	Duration of follow-up post-intervention	Longer-term outcomes	Quality of evidence (GRADE)
Diet/physical activity/behavior (0-6 years) (495)	7	923	−0.40 (−0.85 to 0.05, n = 64)	−0.26 (−0.37 to −0.16, n = 210)	6-12 months	6-12 months	Mean difference BMI −1.00 kg/m^2^ (−1.79 to −0.21) Mean difference BMI SDS −0.25 (−0.40 to −0.10)	Low to very low
Diet/physical activity/behavior (6-11 years) (496)	70	8461	−0.53 (−0.82 to −0.24, n = 2785)	−0.06 (−0.10 to −0.02, n = 4019)	10 days-2 years	1 month–2 years	Mean difference BMI −0.07 kg/m^2^ (−0.34 to 0.20) Mean difference BMI SDS −0.01 (−0.06 to 0.03)	Low to very low
Diet/physical activity/behavior (12-17 years) (497)	44	4781	−1.18 (−1.67 to −0.69, n = 2774)	−0.13 (−0.21 to −0.05, n = 2399)	6 weeks-2 years	6 months-2 years*^a^*	Mean difference BMI −1.49 kg/m^2^ (−3.95 to 0.96) Mean difference BMI SDS −0.15 (−0.21 to −0.09)	Moderate to low
Sibutramine (498)	5	568	−1.70 (−2.89 to −0.51, n = 568)	Not reported	3-12 months	6-13 months*^a^*	Not reported	Low
Metformin (498)	8	543	−1.35 (−2.00 to −0.69, n = 543)	Not reported	3-6 months	6 months-100 weeks*^a^*	Not reported	Low
Orlistat (498)	3	773	−0.79 (−1.08 to −0.51, n = 773	Not reported	6-12 months	5-15 months*^a^*	Not reported	Low
Laparoscopic adjustable gastric band (499, 500)	1	50	−11.4 (not reported)	−0.85 (not reported)	N/A	24 months	Not reported	Low

^*a*^Unclear how duration of follow-up relates to end of intervention.

Drug treatments have also been trialed in the treatment of pediatric obesity, including sibutramine (a 5-HT and noradrenaline reuptake inhibitor), orlistat (a lipase inhibitor) and metformin (a biguanide capable of activating adenosine monophosphate-activated protein kinase (AMPK), which increases insulin sensitivity). Of these a meta-analysis demonstrated that sibutramine showed the biggest overall effect on BMI reduction (mean difference −1.70 kg/m^2^ (95% CI −2.89 to −0.51), *P* < .00001), but this drug has been withdrawn from both European and US markets due to adverse cardiovascular events (498, 501). The use of metformin outside the setting of type 2 diabetes was shown in the same analysis to lead to a significant reduction in BMI as well (mean difference −1.35 kg/m^2^ (95% CI −2.00 to −0.69), *P* < .0001) at up to nearly 2 years follow-up, but concordance is often hampered by gastrointestinal side effects.

Bariatric surgical interventions are now commonplace in the treatment of adult common obesity, but their use in the pediatric setting has not been subject to sufficient trials and may not be ethically justifiable. The most recent Cochrane meta-analysis only included 1 RCT of laparoscopic adjustable gastric banding (LAGB) in patients <18 years old, which recorded a BMI SDS reduction of −1.08 (95% CI −1.31 to −0.86) in the LAGB group vs −0.23 (95% CI −0.05 to 0.39) in the control group receiving lifestyle intervention alone (ΔBMI −12.7 kg/m^2^ [95% CI −14.3 to −11.3] vs −1.3 kg/m^2^ [95% CI −0.4 to −2.9]) (499, 500). Additionally, all participants in the former group had complete reversal of their metabolic syndrome at 24 months’ follow-up. A more recent review confirmed the efficacy of bariatric surgery in adolescents, with mean BMI reductions ranging from −15.0 kg/m^2^ (95% CI −16.5 to −13.5) with gastric bypass surgery to −10.3 kg/m^2^ (95% CI −13.7 to 7.0) with gastric banding at 3 years’ follow-up (502).

Bariatric surgery has long demonstrated excellent results for adult obesity, with a meta-analysis showing sustained weight loss (mean ΔBMI ranging from −7.4 to −33.31 kg/m^2^ vs −0.5 to −4.73 kg/m^2^ with lifestyle and/or medical management) over a period of up to 10 years (503). Importantly, this was associated with a reduction in type 2 diabetes, hypertension, hyperlipidemia, the metabolic syndrome, and cardiovascular events (503, 504). Unlike lifestyle and medical interventions, bariatric surgery resets the energy balance via several endocrine mechanisms, including an increase in insulin secretion and sensitivity (by increasing adiponectin and GLP-1 secretion and *INSR* expression), as well as an increase in anorexigenic pathway (by increasing GLP-1 and PYY secretion) with a concomitant reduction in orexigenic pathway signaling (by reducing ghrelin secretion) (505, 506). This would be in keeping with evidence from several meta-analyses demonstrating its efficacy is positively correlated with the degree of surgical irreversibility (mean difference in BMI at follow-up, laparoscopic gastric bypass vs LAGB −5.21 kg/m^2^ (95% CI −6.39 to −4.03) (503, 507, 508). Some studies, however, demonstrate a waning of efficacy over time, and this “rebound” may occur more quickly in adolescents (502).

### Current Management Strategies for HyOb

Given the current relative lack of efficacious lifestyle interventions and medical treatments for common obesity, it is therefore not surprising that the vast majority of treatment options that have been trialed in HyOb demonstrate only a maintenance of BMI or insignificant, nonsustained weight loss (Table 3). Furthermore, the vast majority of these interventional studies have been limited to case reports, small case series, or uncontrolled cohorts with relatively short durations of follow-up.

**Table 3.** Interventional studies of HyOb in humans

Intervention	Design	Etiology of HyOb	Dose	Number of participants, n	Weight parameter change	Follow-up duration	Adverse events/comments
Truncal vagotomy (509)	Case report	Craniopharyngioma	—	1	Δweight −7 kg	3 years	Delayed gastric emptying
CCK-8 (510)	Cohort (uncontrolled)	Hypothalamic tumor and/or hypothalamic neurosurgery	69 ng/kg IV over 15 minutes	5	Not reported (reduced appetite)	1 day	Gastrointestinal symptoms
Fluoxetine and fenfluramine (511)	Case report	Optic pathway glioma	Fluoxetine 20 mg/day for 1 week, 60 mg/day for 3 months Fenfluramine 60 mg/day for 6 weeks	1	Fluoxetine: Δweight +3 kg Fenfluramine: Δweight 0 kg	Fluoxetine: 3 months Fenfluramine: 6 weeks	None reported
Octreotide (512)	Cohort (uncontrolled)	Hypothalamic tumor and/or hypothalamic neurosurgery/radiotherapy	5-15 μg/kg/day SC	9	Mean ΔBMI −2.0 ± 0.7 kg/m^2^	6 months	Abdominal discomfort, diarrhea, gallstones, reduced GH and free T_4_
Liothyronine (T_3_) (513)	Case series	Hypothalamic tumor and/or hypothalamic neurosurgery/radiotherapy	20-60 μg/day	3	ΔBMI range −6.4 to −5 kg/m^2^	11-27 months	Biochemical hyperthyroidism
Dextroamphetamine (514)	Case series	Craniopharyngioma	12.5-20 mg/day	5	ΔBMI −3 to +0.3 kg/m^2^	2 years	Headaches; note increased physical activity, reduced hyperactivity and improved sleep
Octreotide (515)	RCT	Hypothalamic tumor and/or hypothalamic neurosurgery/radiotherapy	5-15 μg/kg/day SC	9 (9 placebo)	Mean ΔBMI −0.2 ± 0.2 kg/m^2^ (vs placebo +2.2 ± 0.5 kg/m^2^)	6 months	Abdominal discomfort, diarrhea, gallstones, diabetes mellitus
Octreotide (322)	Cohort (uncontrolled)	Prader–Willi syndrome	5 μg/kg/day SC	4	No change	5-7 days	Diarrhea
Octreotide & laparoscopic gastric bypass surgery + truncal vagotomy (516)	Case report	Craniopharyngioma	5 μg/kg-1000 μg/day SC	1	Octreotide: Δweight no change Gastric bypass: Δweight −49 kg	Octreotide 19 months Gastric bypass: 2.5 years	Iron deficiency anemia
Sibutramine (517)	RCT	Mixed group, including postneurosurgery for hypothalamic tumor, Prader–Willi syndrome, Bardet–Biedl syndrome, MC4R mutation	10-15 mg/day PO	19 (26 common obesity controls)	Whole group mean ΔBMI SDS −0.7 (after 20 weeks) Whole group total mean ΔBMI SDS (for continuous 48 week sibutramine group) −1.0	20 RCT + 28 open-label weeks	None; note HyOb patients were more resistant to effects (estimated ΔBMI SDS −0.2)
Caffeine + ephedrine (518)	Case series	Hypothalamic tumor and hypothalamic neurosurgery	Caffeine 600-1200 mg/day PO + ephedrine 75-150 mg/day PO	3	Δweight range −18.8% to −8%	6 months-6 years	Shakiness; note in addition to caloric restriction
Gastric bypass surgery (519)	Case report	Craniopharyngioma	—	1	ΔBMI −16.6 kg/m^2^	18 months	None; note started on vitamin and mineral supplementation prophylactically
Gastric bypass surgery (520)	Case report	Suprasellar mass	—	1	ΔBMI −22 kg/m^2^	4 years	Fibromyalgia, diarrhea, nonhypoglycemic dumping syndrome, hyperuricemia, acute gallstone pancreatitis
Biliopancreatic diversion with duodenal switch procedure (520)	Case report	Suprasellar mass	—	1	ΔBMI −8 kg/m^2^	2 years	Bradycardia, intestinal stenosis
Lifestyle modification (comprehensive care clinic model involving dietary and physical activity advice, behavioral therapy and metformin) (521)	Cohort	Hypothalamic tumor and/or hypothalamic neurosurgery/radiotherapy	—	39	Median ΔBMI rate +4.5 kg/m^2^/year (range −17.8 to +8.4) Median ΔBMI SDS rate 0.0/year (−5.2 to 0.5)	3-41 months	None; note median ΔBMI rate was significantly lower than prior to comprehensive care clinic (median +8.4 kg/m^2^/year (range −3.1 to 28.1))
LAGB (522)	Case series	Craniopharyngioma	—	4	ΔBMI +1.7 to +8.7 kg/m^2^ ΔBMI SDS −1.2 to +3.5	5.3-9.1 years	None
Diazoxide and metformin (523)	Cohort (uncontrolled)	Craniopharyngioma	Diazoxide 2 mg/kg/day PO Metformin 2 g/day PO	9	Mean ΔBMI −0.3 ± 2.3 kg/m^2^ Mean ΔBMI SDS −0.04 ± 0.15	6 months	Oedema, deranged liver enzymes, vomiting
Gastric bypass surgery (524)	Case report	Craniopharyngioma	—	1	ΔBMI −12.6 kg/m^2^	19 months	None
Exenatide/liraglutide (525)	Case report	Hypothalamic tumor and radiotherapy	Not reported	1	Δweight −10 kg (−6.7%)	4 years	Nausea; note in combination with insulin and gliclazide
Exenatide (526)	Case report	Germ cell tumor	10 μg/day SC	1	ΔBMI −10.2 kg/m^2^	2.5 years	None; note in combination with metformin
LAGB/sleeve gastrectomy/gastric bypass surgery (527)	Case-control	Craniopharyngioma	—	9 (143 common obesity controls)	LAGB (n = 6): No change Sleeve gastrectomy (n = 4): No change Gastric bypass (n = 2): Similar to controls mean Δweight −30%	LAGB: 1-9 years Sleeve gastrectomy: 0.5-4 years Gastric bypass: 2-4 years	Abdominal pain, vomiting, gastro-esophageal reflux, impaired absorption of desmopressin, adrenal crisis; note HyOb patients more resistant to LAGB and sleeve gastrectomy compared to common obesity controls
Sleeve gastrectomy/gastric bypass surgery (528)	Case series	Hypothalamic tumor and/or hypothalamic neurosurgery ± radiotherapy	—	4	Sleeve gastrectomy (n = 2): ΔBMI range −10 to −3.6 kg/m^2^ Gastric bypass (n = 2): ΔBMI range −6.2 to +11.3 kg/m^2^	Sleeve gastrectomy: 2.5 years Gastric bypass: 2-5.5 years	None; note in combination with metformin, sitagliptin, insulin, GLP-1 agonists, and/or sulfonylureas
Vertical ring gastroplasty (529)	Case report	*LEPR* mutation	—	1	LAGB: Δweight −46 kg (−28%) Vertical ring gastroplasty: Δweight −40 kg (−20%)	LAGB: 1 year Vertical ring gastroplasty: 8 years	LAGB: Gastric band slippage Vertical ring gastroplasty: None
Exenatide/liraglutide (530, 531)	Case series	Hypothalamic tumor and/or hypothalamic neurosurgery/radiotherapy	Exenatide 5-10 μg/day SC Liraglutide 0.6 mg/day SC	9	ΔBMI −6.1 to −2.8 kg/m^2^ Δweight −22 to −9 kg	6-51 months	Nausea, vomiting
Liraglutide (531)	Case series	Traumatic brain injury, meningitis	0.3–0.9 mg/day SC	2	Δweight −11 to −3 kg	18 months—2 years	None
Liothyronine (T_3_) (532)	Case report	Craniopharyngioma	37.5 μg/day PO	1	No change	2 months	Biochemical hyperthyroidism
Exenatide (533)	Cohort (uncontrolled)	Hypothalamic tumor and/or hypothalamic neurosurgery/radiotherapy	10–20 μg/day SC	8	Mean Δweight −1.4 kg	50 weeks	Nausea, vomiting, joint pain, injection site reactions; note change in weight not statistically significant
Bilateral nucleus accumbens deep brain stimulation (DBS) (534)	Case report	Craniopharyngioma	—	1	ΔBMI −5.2 kg/m^2^	14 months	Accidental switching off of pacemaker
Sleeve gastrectomy/gastric bypass surgery (535)	Case-control	Craniopharyngioma	—	8 (75 common obesity controls)	Sleeve gastrectomy (n = 3): mean Δweight −10% Gastric bypass (n = 5): mean Δweight −25%	2 years	Increased GH and desmopressin requirements; note sleeve gastrectomy not efficacious in HyOb, gastric bypass outcomes comparable to common obesity
Sleeve gastrectomy (536)	Case series	Craniopharyngioma	—	3	Mean ΔBMI −13.9 kg/m^2^ Δweight −17.6% to −41.1%	2 years	Increased GH requirements; note reduction in levothyroxine and desmopressin requirement
Beloranib (537)	RCT	Craniopharyngioma, pituitary macroadenoma	1.8 mg twice weekly SC	8 (6 placebo)	Mean Δweight −6.2 kg (−3.4 kg vs placebo −0.3 kg at 4 weeks)	4 RCT + 4 open label weeks	Urticaria, dizziness, gastroenteritis, neutropenia; note placebo group achieved similar degree of weight loss in 4-week open label phase
OXT and naltrexone (538)	Case report	Craniopharyngioma	OXT 6 IU every 3 days-daily IN Naltrexone 100 mg/day PO	1	OXT: ΔBMI SDS −0.28 OXT + naltrexone: ΔBMI SDS −0.67	10 weeks 38 weeks	None
Dextroamphetamine (539)	Cohort (uncontrolled)	Craniopharyngioma, low-grade glioma, meningitis, pseudohypoparathyroidism type 1a, Bardet–Biedl syndrome, *LEPR* deficiency, Prader–Willi-like syndrome, Temple syndrome, congenital hydrocephalus (Chiari II malformation	5-40 mg/day	19	ΔBMI SDS −0.14 (11 declined, 3 stabilized)	23.7 ± 12.7 months	Hypertension, sleep difficulties, change in emotional state
Tesomet (tesofensine + metoprolol) (540)	RCT	Craniopharyngioma, pituitary macroadenoma, astrocytoma, meningioma, glioma, germinoma	0.5 mg tesofensine/50 mg metoprolol	18	Mean additional Δweight change −6.3% (tesomet −6.6% vs placebo −0.3%) % patients achieving >5% weight reduction Tesomet 61.5% vs placebo 12.5%	24 weeks	Sleep disturbance, dry mouth, headache, dizziness

The first interventional trial for HyOb in humans was based on the theory of hyperinsulinemia being the primary driver of weight gain, where a young adult with a craniopharyngioma, panhypopituitarism and HyOb underwent a truncal vagotomy, which led to a degree of sustained weight loss, but with the side effect of delayed gastric emptying (509). More than 10 years later, similar studies of medical treatment for hyperinsulinemia using octreotide (a SS analogue) in children with HyOb resulted in a minimal reduction in BMI in some patients (mean BMI reduction of −0.2 kg/m^2^ in an RCT setting), but with the side effects of diarrhea, abdominal discomfort, cholelithiasis, diabetes mellitus, and impairment of the GH and TSH hypothalamo-pituitary axes (322, 512, 515). A similar strategy, using a combination of diazoxide (to reduce hyperinsulinemia) and metformin (to increase insulin sensitivity) also achieved relatively minimal results, but with the side effects of oedema and liver dysfunction (523). It is important to note that none of these groups reported further success in larger cohorts with longer durations of follow-up beyond 6 months. Dysregulation of other appetite-regulating hormones is also a potential side effect of some of these therapies, thereby making their effects more unpredictable (gut hormones with pancreatic vagotomy; GH, TSH, CCK, glucagon, GLP-1, PPY, ghrelin, and gastrin with octreotide).

Numerous other medical therapies have been tried in HyOb, none of which have yet demonstrated long-term sustainable weight loss. Of note, several authors have trialed GLP-1 agonists (exenatide and liraglutide) in individual or small groups of patients with apparently impressive results (maximum BMI reduction −10.2 kg/m^2^ at 2.5-year follow-up), but a RCT is sorely needed to confirm these findings, particularly as tolerability is variable due to nausea and vomiting being common side effects (525, 526, 530, 531, 533). Other medications such as CCK-8 (510), liothyronine (513), fluoxetine and fenfluramine (511), dextroamphetamine (514, 539), sibutramine (517), caffeine with ephedrine (518), and beloranib (537) have been tried, with no major breakthroughs, or unacceptable side effects (both sibutramine and beloranib have been withdrawn from use). Most recently, 2 phase 2 trials have shown promising results for hypothalamic obesity. A randomized controlled trial using a combination of tesofensine (a monoamine reuptake inhibitor with a better pharmacokinetic profile than sibutramine) and metoprolol resulted in an additional −6.3% mean weight loss over 6 months (540). Currently an open label single arm phase 2 trial of setmelanotide is in progress, having reported positive interim results with a −17.2% mean BMI reduction in 4 months (n = 11) (541). Contrastingly, modification of lifestyle behaviors alone achieved a slowing of weight gain rather than weight loss per se (521).

As with common obesity, the most impressive weight reductions have been achieved with bariatric surgery (maximum BMI reduction −22 kg/m^2^ at 4 years) (520). However, the effects of bariatric surgery are more unpredictable, and it is apparent that patients with HyOb can be more resistant, particularly to less radical procedures such as LAGB or sleeve gastrectomies (522, 527, 535). Experience with bariatric surgery in HyOb has also been more limited and not subject to the same durations of follow-up as in common obesity—the most longstanding procedure, LAGB, has shown no effect on weight, or indeed, even weight gain, over a period of 9 years (522, 527). Additionally, on top of the cardiorespiratory risks of administering general anesthesia in obesity, patients with HyOb usually have substantial comorbidities such as hypopituitarism (particularly adrenal insufficiency and central diabetes insipidus). Several groups have reported an association with changing requirements of hormone supplementation as well as impaired absorption of crucial medications such as desmopressin postoperatively (527, 535, 536). Lastly, there are significant risks of a significantly increased postoperative food intake (due to hyperphagia) in the presence of a reduced gastric capacity.

## Conclusions

Against a background of a rising prevalence in childhood obesity, there is an accruing cohort of patients with various congenital and acquired hypothalamic conditions who are at risk of developing HyOb. To date, our understanding of the pathophysiology of both common obesity and HyOb remains incomplete, but the advent of newer molecular genetic techniques has permitted the discovery of the complex and often redundant network of peptides governing energy homeostasis between the gastrointestinal tract, adipose tissue and hypothalamus. These discoveries have consequently led to the development of targeted molecular therapies for specific monogenic HyOb syndromes. However, none of these treatments have so far been proven efficacious in the treatment of other congenital and acquired syndromic forms of HyOb as it is likely that more than 1 component of the appetite- and weight-regulating neurocircuitry may be affected. Ultimately, in this more heterogenous group of disorders, a clearer understanding of these pathways and how this influences the individualized treatment options in a particular patient is still much needed.

## Abbreviations

5-HT: serotonin
αMSH: α-melanocyte stimulating hormone
ACTH: adrenocorticotropic hormone
AgRP: Agouti-related peptide
ARC: arcuate nucleus
AVP: arginine–vasopressin
BDNF: brain-derived neurotrophic factor
BMI: body mass index
CART: cocaine- and amphetamine-regulated transcript
CCK: cholecystokinin
CNS: central nervous system
CRH: corticotrophin-releasing hormone
DMN: dorsomedial nucleus
DPP4: dipeptidyl peptidase-4
FOXO1: Forkhead box protein 1
FSH: follicle-stimulating hormone
GH: growth hormone
GHRH: growth hormone-releasing hormone
GHSR: growth hormone secretagogue receptor
GLP-1: glucagon-like peptide-1
GOAT: ghrelin-O-acyltransferase
HCRT: hypocretin
HCRTR: hypocretin receptor
HyOb: hypothalamic obesity
IDDM: insulin-dependent diabetes mellitus
INSR: insulin receptor
JAK: Janus kinase
LAGB: laparoscopic adjustable gastric banding
LEPR: leptin receptor
LHA: lateral hypothalamic area
MCH: melanin-concentrating hormone
MCR: melanocortin receptor
NPY: neuropeptide Y
NTS: nucleus tractus solitaries
NUCB2: nucleobindin-2
OXT: oxytocin
PC: prohormone convertase
PI3K: phosphoinositide 3-kinase
PKB: protein kinase B
POMC: proopiomelanocortin
PPY: pancreatic polypeptide Y
PVN: paraventricular nucleus
PYY: polypeptide YY
RCT: randomized controlled trial
REE: resting energy expenditure
SOD: septo-optic dysplasia
SON: supraoptic nucleus
SS: somatostatin
STAT: signal transducer and activator of transcription
TRH: thyrotrophin-releasing hormone
TRKB: tyrosine kinase receptor B
VMA: vanillylmandelic acid
VMN: ventromedial nucleus
